# Convergent Synthesis of Two Fluorescent Ebselen-Coumarin Heterodimers

**DOI:** 10.3390/ph9030043

**Published:** 2016-07-08

**Authors:** Jim Küppers, Anna Christina Schulz-Fincke, Jerzy Palus, Mirosław Giurg, Jacek Skarżewski, Michael Gütschow

**Affiliations:** 1 Pharmaceutical Institute, Pharmaceutical Chemistry I, University of Bonn, An der Immenburg 4, D-53121 Bonn, Germany; jim.kueppers@uni-bonn.de (J.K.); anna.schulz-fincke@uni-bonn.de (A.C.S.-F.); 2 Department of Organic Chemistry, Wrocław University of Technology (A2), Wyspiańskiego 27, 50-370 Wrocław, Poland; jerzy.palus@pwr.wroc.pl (J.P.); miroslaw.giurg@pwr.wroc.pl (M.Gi.); jacek.skarzewski@pwr.wroc.pl (J.S.)

**Keywords:** coumarin, ebselen, heterodimer

## Abstract

The organo-seleniumdrug ebselen exhibits a wide range of pharmacological effects that are predominantly due to its interference with redox systems catalyzed by seleno enzymes, e.g., glutathione peroxidase and thioredoxin reductase. Moreover, ebselen can covalently interact with thiol groups of several enzymes. According to its pleiotropic mode of action, ebselen has been investigated in clinical trials for the prevention and treatment of different ailments. Fluorescence-labeled probes containing ebselen are expected to be suitable for further biological and medicinal studies. We therefore designed and synthesized two coumarin-tagged activity-based probes bearing the ebselen warhead. The heterodimers differ by the nature of the spacer structure, for which—in the second compound—a PEG/two-amide spacer was introduced. The interaction of this probe and of ebselen with two cysteine proteases was investigated.

## 1. Introduction

Ebselen represents a lipid-soluble, selenium-containing, multifunctional drug with a broad range of pharmacological effects including both beneficial and harmful actions. The general mechanism of action is mainly based on reactions with specific thiol groups. This reactivity makes it a potent modulator for proteins that require cysteine for normal function [1,2]. Ebselen has been shown to be an efficient antioxidantin vivo. It was considered to be a relatively nontoxic compound because its selenium is not bioavailable [1,2,3,4]. However, it can act detrimentally through the depletion of glutathione [5,6]. Ebselen targets a wide variety of enzymes and modulates several biological processes. The inhibition of enzymes is based on the high reactivity of ebselen with critical protein thiol groups, leading to the reversible formation of relatively stable seleno-sulfide bonds [1,3]. However, such formation can be reversed by the addition of reducing agents [1], as has been shown, for example, for cerebral Na^+^, K^+^-ATPase and indoleamine 2,3-dioxygenase [7,8]. Ebselen exhibits anti-inflammatory effects due to its ability to directly inhibit inflammation-related enzymes [1,3,9].

The drug ebselen interferes with certain selenoenzymes, an important class of antioxidant biocatalysts that include glutathione peroxidase (GPx) and thioredoxin reductase (TrxR). Glutathione peroxidase protects biomembranes and other cellular components by using glutathione as the reducing substrate for the detoxification of a variety of hydroperoxides. Thioredoxin reductase catalyzes the reduction of thioredoxin (Trx) with NADPH as the cofactor. These transformations create a basis for a number of processes, such as defense against oxidative stress, the synthesis of desoxyribonucleotides, redox regulation of gene expression, and signal transduction [5,10,11]. Ebselen, on the one hand, mimics GPx activity by catalyzing the reduction of peroxides with glutathione, and, on the other hand, has been demonstrated to be an excellent substrate for human TrxR [1,9,12]. As an antioxidant compound and a GPx mimic, ebselen appears to be a potential drug for the treatment of several disorders including diabetes-related diseases associated with reduced GPx levels such as atherosclerosis and nephropathy as well as a prospective treatment for cerebral ischemia. Hence, ebselen has been used in clinical trials for the prevention and treatment of different disorders [1,3].

**Figure 1:**
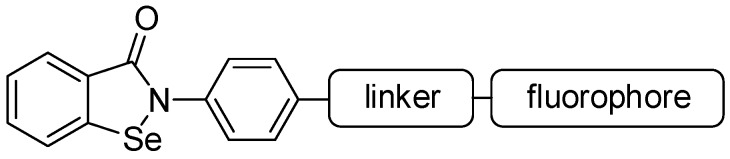
Structure of the fluorescently labeled ebselen derivatives.

In order to provide tool compounds for the ongoing scientific efforts to further characterize the biological activity of ebselen, we designed two activity-based probes containing the intact ebselen structure and a rigidified 7-amino coumarin (coumarin 343) as the fluorescent tag. Coumarins represent a widely used type of fluorophores and are characterized by their small molecular size, large Stokes shifts as well as chemical and enzymatic stability [13,14,15,16,17,18,19,20,21,22,23,24,25,26,27]. In the second heterodimer, the coumarin tag should be connected via a PEG/two-amide linker with the ebselen substructure (Figure 1). It was the aim of this study to synthesize these probes as biochemical tools for future studies. Moreover, we investigated the interaction of both ebselen and the second probe with two model cysteine proteases, i.e., the human cathepsins B and L.

## 2. Results and Discussion

**Scheme 1:**
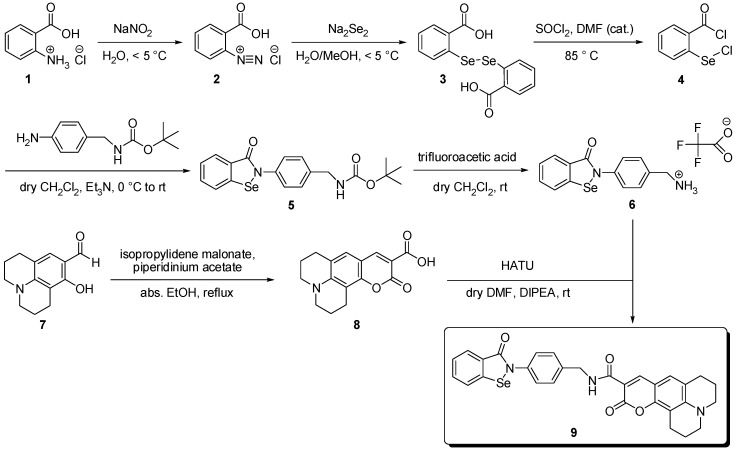
Synthetic route to the coumarin-labeled ebselen derivative **9**.

The two convergent synthetic routes start with the procedure for synthesizing ebselen (2-phenyl-1,2-benzoselenazol-3-one) [28] (Scheme 1). Anthranilic acid (**1**) as the starting material was converted into a diazonium salt **2**, which was treated with a disodium diselenide containing solution to obtain 2,2’-diselenobisbenzoate (**3**). The diselenide required for this step was obtained through a hydrazine-promoted reduction of selenium. After reacting **3** with thionyl chloride and a catalytic amount of DMF, Boc-semiprotected 4-aminomethylaniline was added. The resulting intermediate **5** was deprotected with trifluoroacetic acid to give the ebselen building block **6**. The fluorescent coumarin 343 (**8**) was synthesized by submitting 8-hydroxyjulolidine-9-carboxaldehyde to a Knoevenagel condensation [20]. This was further coupled with building block **6** by using HATU in DMF to yield the first heterodimeric ebselen-coumarin probe **9**.

**Scheme 2:**
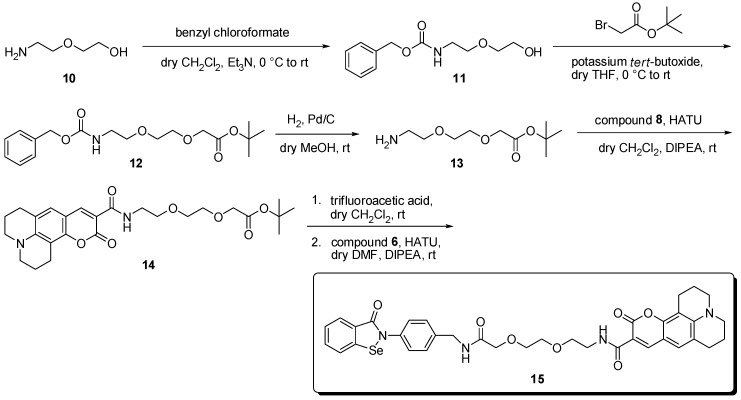
Synthetic route to the coumarin-labeled ebselen derivative **15**.

To incorporate a PEG linker between the ebselen and coumarin substructures, we successfully employed a synthetic strategy in which the linker was first connected with the coumarin and the product was subsequently finalized by introducing the ebselen building block (Scheme 2). To generate the linker structure **13** [20], the amino group of 2-(2-aminoethoxy)ethanol (**10**) was Cbz-protected with benzyl chloroformate, then alkylated at the hydroxy group with *tert*-butyl bromoacetate using potassium *tert*-butoxide as a base to give compound **12** [29]. Afterwards, the linker structure **13** was achieved by a Cbz deprotection with hydrogen and palladium/carbon [20]. The fluorophore **8** was then reacted with **13** by using HATU in dichloromethane. The resulting compound **14** was deprotected with trifluoroacetic acid and finally coupled to the ebselen building block **6** by means of HATU in DMF to assemble the second probe **15**.

**Figure 2:**
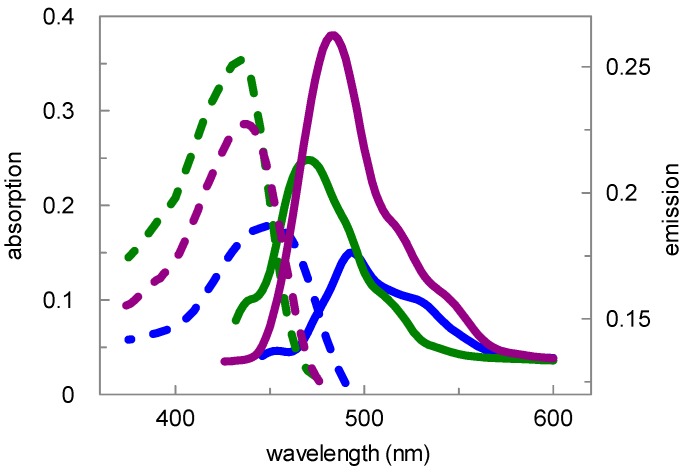
Absorption (10 μM, 1% DMSO, dashed lines) and emission (1 μM, 1% DMSO, solid lines) spectra of the ebselen-coumarin heterodimer **15**. The extinction and the fluorescence units, respectively, were plotted versus the wavelength. Spectra recorded in buffer (50 mM sodium phosphate, pH 7.8, with 500 mM NaCl) (**blue**), MeOH (**violet**) and CH_2_Cl_2_ (**green**) are shown. Wavelengths of absorption were used for excitation. The heterodimer **9** was measured similarly (see Table 1). All fluorescent measurements were carried out with a PMT value of 200 V.

The structures of the new compounds were confirmed by analytical data, including high-resolution mass spectra (HRMS) which gave two main signals according to the two predominant isotopes of selenium. We have recorded UV/Vis and fluorescence spectra of **9** and **15** in three different solvents. Coumarins exhibit advantageous features, i.e. a high fluorescent intensity and a large Stokes shift. Such properties were also obtained for our heterodimers, as shown in Figure 2 and Table 1.

Ebselen was reported to react with its targets through the formation of covalent seleno-sulfide bonds. This covalent mode of interaction provides an impetus for the development of activity-based probes. Recently, an ebselen-cyanine probe was reported to be utilized for the real-time imaging of the cellular redox status changes [30]. In this study, we have coupled the ebselen warhead with coumarin 343. The fluorophor coumarin 343 is valued for its bathochromic shift of absorption and emission and its high fluorescence quantum yield, even in aqueous medium [31]. These desirable properties arise from its rigidified tetracyclic structure. Compared to less rigid 7-donor substituted coumarins, the rotation of the amino group in coumarin 343 is constrained and the nitrogen lone pair can maximally interact with the aromatic system [32,33]. Accordingly, coumarin 343 was incorporated as a fluorescence label in various activity-based probes [13,18,20].

Besides the direct connection of the ebselen structure with that of coumarin 343, realized in compound **9**, we have designed the second probe **15** comprising a PEG/two-amide spacer. The incorporation of this flexible spacer is thought to facilitate the interaction of the ebselen part with putative targets by preventing a possible steric hindrance caused by the coumarin part of the heterodimer. Moreover, **15** was unsurprisingly found to be more soluble than **9** in different organic solvents. Compounds **9** and **15** expand the portfolio of ebselen homo- and heterodimers [2,4,12,30,34,35]. Our ebselen containing fluorescence-labeled probes are expected to be suitable pharmacological tools to continuously elucidate the biological activity of ebselen.

The known reactivity of ebselen with cysteine residues of several proteins [1,7,8,36,37,38] prompted us to investigate its interaction with two model cysteine proteases, the human cathepsins B and L. An inactivation of these enzymes through a seleno-sulfide bond formation would provide a possible starting point for the development of probes for cysteine proteases. Ebselen and probe **15** were added at a final concentration of 10 μM to the cathepsin activity assays [39,40]. Duplicate experiments revealed no inhibition of the proteolytic activity by using peptidic chromogenic substrates. However, the covalent interaction of ebselen and assumedly of **15** with critical protein thiol groups can be reversed by the addition of reducing compounds, such as dithiothreitol (DTT) [1,8,36,37]. In our usual cathepsin measurements, DTT is applied for the activation of the cathepsins, leading to rather high final DTT concentrations of 100 μM (cathepsin B) and 200 μM (cathepsin L) in the assays. Hence, the strong excess of DTT might prevent enzyme inhibition by the two compounds. We have modified the assays as follows: (i) the final DTT concentration was reduced to 3.5 μM; (ii) the final DMSO concentration was changed from 2% to 5%; (iii) the enzymes were preincubated for 10 min with the substrate prior to the addition of the test compounds; and (iv) the final enzyme concentration was increased 3.5-fold (cathepsin B) and 1.75-fold (cathepsin L). Under such conditions, the enzymes could be assayed appropriately with the decreased amount of DTT. Ebselen and probe **15** were investigated at final concentrations of 1 μM and 0.2 μM in duplicate measurements. Unexpectedly, the proteolytic activity was stimulated, in particular that of cathepsin B, when each of the two compounds was added. Obviously, the test compounds were able to interfere with the redox equilibrium between the protein and DTT. It could therefore be concluded that probe **15** is not suitable for labeling cysteine cathepsins. Nevertheless, the fluorescent ebselen derivatives might be helpful to identify proteins which are targeted by this drug and might therefore be valuable in future biological studies.

## 3. Materials and Methods

### 3.1. General Methods and Materials

Thin-layer chromatography was carried out on Merck aluminum sheets, silica gel 60 F254. Detection was performed with UV light at 254 nm. Preparative column chromatography was performed on Merck silica gel 60 (70–230 mesh). Melting points were determined on a Büchi 510 oil bath apparatus (Büchi, Essen, Germany). Mass spectra were recorded on an API 2000 mass spectrometer (electron spray ion source, Applied Biosystems, Darmstadt, Germany) coupled with an Agilent 1100 HPLC system (Agilent Technologies, Santa Clara, CA, USA) using a Phenomenex Luna HPLC C_18_ column (50 × 2.00 mm, particle size 3 μm).

The purity of compounds was determined by HPLC-DAD obtained on an LC-MS instrument (HPLC Agilent 1100). HRMS was recorded on a microTOF-Q mass spectrometer (Bruker, Köln, Germany) with ESI source coupled with a HPLC Dionex Ultimate 3000 (Thermo Scientific, Braunschweig, Germany) using a EC50/2 Nucleodur C18 Gravity 3 μm column (Macherey-Nagel, Düren, Germany). ^1^H-NMR (500 MHz) and ^13^C-NMR (126 MHz) were recorded on a Bruker Avance DRX 500 and ^1^H-NMR (600 MHz) and ^13^C-NMR (151 MHz) spectra on a Bruker Avance III 600. Chemical shifts δ are given in ppm referring to the signal center using the solvent peaks for reference: DMSO-*d*_6_ 2.49/39.7 ppm. Benzyl 2-(2-hydroxyethoxy)ethylcarbamate (**11**), *N*-(benzyloxycarbonyl)-2-(2-(2-aminoethoxy)-ethoxy)acetate *tert*-butyl ester (**12**) [29] and *tert*-butyl 2-(2-(2-aminoethoxy)ethoxy)acetate (**13**) were prepared as described [20]. UV spectra were recorded on a Cary 50 Bio, Varian spectrophotometer (Agilent Technologies, Santa Clara, CA, USA). Fluorescence spectra were recorded on a Safas Monaco spectrofluorometer flx (Monaco, Principality of Monaco).

### 3.2. Syntheses

*2,2′-Diselenobisbenzoic acid* (**3**). A disodium diselenide solution was prepared by the reaction of selenium powder (5.9 g, 75 mmol), 100% hydrazine hydrate (0.98 g, 0.95 mL, 20 mmol) and sodium hydroxide (9 g, 22.5 mmol) in MeOH (150 mL) carried out at rt for 24 h. Meanwhile, to a stirred solution of anthranilic acid (**1**, 10.3 g, 75 mmol) in 3N HCl (60 mL) cooled with an ice/salt bath, a solution of sodium nitrite (5.7 g, 83 mmol) in water (15 mL) was added dropwise, and the temperature was maintained below 5 °C. After stirring for additional 15 min, this solution was added dropwise to the stirred solution containing disodium diselenide cooled in an ice/salt bath below −5 °C. The temperature was kept for 2.5 h and then increased to rt for another 20 h. The reaction mixture was filtrated and the filtrate was acidified to pH 3 by adding 3N HCl (300 mL). After 30 min, the obtained precipitate was filtered off, washed with hot water (2 L), dried at 90 °C for 4 h, then on air for another 24 h, and recrystallized from 1,4-dioxane resulting in the pure product **3** (2.7 g, 18%); m.p. > 250 °C, lit. [41] m.p. 297 °C, decomp; ^1^H-NMR (600 MHz, DMSO-*d*_6_) δ 13.68 (s, 2H, CO_2_H), 8.02 (dd, *J* = 7.7, 1.6 Hz, 2H, 2 × 6-H), 7.67 (dd, *J* = 8.3, 1.2 Hz, 2H, 2 × 3-H), 7.48 (ddd, *J* = 8.3, 7.2, 1.6 Hz, 2H, 2 × Ar-H), 7.35 (td, *J* = 7.4, 1.2 Hz, 2H, 2 × Ar-H); ^13^C-NMR (151 MHz, DMSO-*d*_6_) δ 168.7, 133.7, 133.6, 131.7, 129.7, 128.9, 126.7; LC-MS(ESI) (90% H_2_O to 100% MeOH in 10 min, then 100% MeOH over 10 min, DAD 200–400 nm), 96% purity, *m/z* = 400.9, 403.0 ([M+H]^+^), 418.0, 420.0 ([M+NH_4_]^+^).

*tert-Butyl 4-(3-oxobenzo[d][1,2]selenazol-2(3H)-yl)benzylcarbamate* (**5**) [34]. Compound **3** (8.0 g, 20 mmol) was added to thionyl chloride (40 mL) and DMF (1 mL), and the reaction mixture was refluxed at 85 °C for 3 h. The solvent was then evaporated, and the obtained product was recrystallized from *n*-hexane resulting in pale yellow prisms of 2-(chloroseleno)benzoyl chloride **4** (4.65 g, 46%); m.p. 64–66 °C, lit. [4] mp 64–66 °C, which was used without further characterization. In the next step, a solution of compound **4** (2.54 g, 10 mmol) in dry CH_2_Cl_2_ (16 mL) was added dropwise to a stirred solution containing 4-[(*N*-Boc)aminomethyl]aniline (1.78 g, 8 mmol) in dry CH_2_Cl_2_ (24 mL) and triethylamine (2.77 mL, 2.02 g, 20 mmol) over a period of 15 min at 0 °C. After stirring at rt for another 24 h, the obtained solid was filtered off and the residue was washed with CH_2_Cl_2_ (2 × 1 mL) to give compound **5** as a white solid (2.49 g, 77%); m.p. 165–168 °C; ^1^H-NMR (500 MHz, DMSO-*d*_6_) δ 8.07 (dt, *J* = 8.1, 0.8 Hz, 1H, 4-H), 7.89 (ddd, *J* = 7.7, 1.4, 0.6 Hz, 1H, 7-H), 7.67 (ddd, *J* = 8.1, 7.2, 1.4 Hz, 1H, Ar-H), 7.59–7.53 (m, 2H, phenylene 2-H, 6-H), 7.49–7.45 (m, 1H, Ar-H), 7.39 (t, *J* = 6.3 Hz, 1H, NH), 7.32–7.27 (m, 2H, phenylene 3-H, 5-H), 4.13 (d, *J* = 6.2 Hz, 2H, CH_2_), 1.40 (s, 9H, C(CH_3_)_3_); ^13^C-NMR (126 MHz, DMSO-*d*_6_) δ 165.1, 155.9, 139.0, 138.4, 138.1, 132.3, 128.6, 128.0, 127.8, 126.4, 125.9, 124.8, 78.0, 43.1, 28.4; LC-MS(ESI) (90% H_2_O to 100% MeOH in 10 min, then 100% MeOH over 10 min, DAD 200–400 nm), 100% purity, *m*/*z* = 403.2, 405.1 ([M+H]^+^), 420.2, 422.1 ([M+NH_4_]^+^); HRMS (ESI): *m*/*z* [M+H]^+^) calcd. for C_19_H_20_N_2_O_3_Se: 403.0720, 405.0712; found: 403.0721, 405.0716.

*(4-(3-Oxobenzo[d][1,2]selenazol-2(3H)-yl)phenyl)methanaminium 2,2,2-trifluoroacetate* (**6**). Compound **5** (403 mg, 1 mmol) was dissolved in dry CH_2_Cl_2_ (30 mL) and trifluoroacetic acid (6 mL) was added. After stirring at rt for 2 h, volatiles were evaporated and the residue was diluted with CH_2_Cl_2_ (4 × 10 mL). The solvent evaporated to remove the excess of trifluoroacetic acid yielding compound **6** as a pale yellow salt (411 mg, 99%); mp 198–201 °C; ^1^H-NMR (500 MHz, DMSO-*d*_6_) δ 8.22 (s, 3H, NH_3_^+^), 8.10 (dt, *J* = 8.1, 0.8 Hz, 1H, 4-H), 7.91–7.88 (m, 1H, 7-H), 7.71–7.66 (m, 3H, Ar-H, phenylene 2-H, 6-H), 7.54–7.50 (m, 2H, phenylene 3-H, 5-H), 7.50–7.46 (m, 1H, Ar-H), 4.06 (q, *J* = 5.8 Hz, 2H, CH_2_); ^13^C-NMR (126 MHz, DMSO-*d*_6_) δ 165.3, 140.2, 139.0, 132.5, 131.4, 130.0, 128.6, 128.1, 126.4, 126.0, 124.7, 42.0; LC-MS(ESI) (90% H_2_O to 100% MeOH in 10 min, then 100% MeOH over 10 min, DAD 200–400 nm), 100% purity, *m/z* = 303.0, 305.1 ([M+H]^+^); HRMS (ESI): *m*/*z* [M+H]^+^) calcd. for C_14_H_12_N_2_OSe: 303.0196, 305.0188; found: 303.0196, 305.0207.

*2,3,6,7-Tetrahydro-11-oxo-1H,5H,11H-[1]benzopyrano-[6,7,8-ij]quinolizine-10-carboxylic Acid, Coumarin 343* (**8**) [20]. 8-Hydroxyjulolidine-9-carboxaldehyde (**7**, 2.17 g, 10.0 mmol) was dissolved in absolute EtOH (10 mL). Isopropylidene malonate (1.44 g, 10.0 mmol) and piperidinium acetate (61 mg, 0.42 mmol) were added to the solution and stirred for 20 min at rt and refluxed for 2 h. The reaction mixture was allowed to cool down to rt and chilled in an ice bath for 30 min. The product was filtered off and washed with EtOH (10 mL) to yield **8** as an orange solid (0.63 g, 22%); m.p. 251–254 °C, lit. [42] m.p. 253 °C; ^1^H-NMR (500 MHz, DMSO-*d*_6_) δ 8.44 (s, 1H, 9-H), 7.22 (s, 1H, 8-H), 3.36–3.32 (m, 4H, N(C*H*_2_CH_2_CH_2_)_2_), 2.73–2.69 (m, 4H, N(CH_2_CH_2_C*H*_2_)_2_), 1.91–1.83 (m, 4H, N(CH_2_C*H*_2_CH_2_)_2_), the CO_2_H signal could not be detected; ^13^C-NMR (126 MHz, DMSO-*d*_6_) δ 164.7, 160.8, 152.8, 149.3, 148.9, 127.6, 119.7, 107.4, 105.2, 104.9, 49.8, 49.3, 26.9, 20.6, 19.6; LC-MS(ESI) (90% H_2_O to 100% MeOH in 10 min, then 100% MeOH over 10 min, DAD 200–500 nm), 98% purity, *m*/*z* = 286.1 ([M+H]^+^).

*Ebselen-coumarin Heterodimer* (**9**). A solution containing compound **8** (143 mg, 0.50 mmol), HATU (228 mg, 0.60 mmol) and DIPEA (0.52 mL, 388 mg, 3 mmol) in DMF (8 mL) was stirred at rt for 30 min. Compound **6**(250 mg, 0.60 mmol) was added and the resulting mixture was stirred for 96 h at rt. The solvent was evaporated to dryness. The residue was redissolved in CH_2_Cl_2_ (50 mL) and washed with aqueous saturated NaHCO_3_ solution (50 mL), aqueous 10% KHSO_4_ solution (50 mL), water (50 mL), aqueous saturated NaHCO_3_ solution (50 mL), aqueous 10% KHSO_4_ solution (50 mL) and brine (50 mL). The organic layer was dried over Na_2_SO_4_, filtrated and evaporated. The crude residue was redissolved in a small volume of DMF and silica gel was added to this solution. The solvent was again evaporated. The crude product attached to silica gel was loaded to a silica gel containing column and purified using CH_2_Cl_2_/ethyl acetate (8:2) as eluent. Corresponding fractions were evaporated to give compound **9** as yellow solid (0.036 g, 13%); m.p. 237–240 °C, decomp.; ^1^H-NMR (500 MHz, DMSO-*d*_6_) δ 9.09 (t, *J* = 6.0 Hz, 1H, NH), 8.54 (s, 1H, coumarin 9-H), 8.08–8.05 (m, 1H, ebselen 4-H), 7.88 (dd, *J* = 7.9, 1.4 Hz, 1H, ebselen 7-H), 7.67 (ddd, *J* = 8.3, 7.2, 1.4 Hz, 1H, ebselen Ar-H), 7.60–7.56 (m, 2H, phenylene 2-H, 6-H), 7.49–7.44 (m, 1H, ebselen Ar-H), 7.41–7.37 (m, 2H, phenylene 3-H, 5-H), 7.25 (s, 1H, coumarin 8-H), 4.53 (d, *J* = 6.0 Hz, 2H, C*H*_2_NH), 3.33–3.30 (m, 4H, N(C*H*_2_CH_2_CH_2_)_2_), 2.75–2.69 (m, 4H, N(CH_2_CH_2_C*H*_2_)_2_), 1.90–1.85 (m, 4H, N(CH_2_C*H*_2_CH_2_)_2_); ^13^C-NMR (126 MHz, DMSO-*d*_6_) δ 165.1, 162.7, 162.0, 152.3, 148.2, 147.8, 139.0, 138.6, 137.3, 132.3, 128.6, 128.3, 128.0, 127.3, 126.3, 125.9, 124.8, 119.6, 108.0, 107.6, 104.8, 49.7, 49.2, 42.3, 26.9, 20.7, 19.7; LC-MS(ESI) (90% H_2_O to 100% MeOH in 10 min, then 100% MeOH over 10 min, DAD 200–500 nm), 96% purity, *m/z* = 570.1, 572.1 ([M+H]^+^); HRMS (ESI): *m*/*z* [M+H]^+^) calcd. for C_30_H_25_N_3_O_4_Se: 570.1091, 572.1084; found: 570.1043, 572.1032.

*tert-Butyl N-[(2,3,6,7-tetrahydro-11-oxo-1H,5H,11H-[1]benzopyrano-[6,7,8-ij]quinolizin-10-yl)-carbonyl]-2-(2-(2-aminoethoxy)ethoxy)acetate* (**14**) [20]. A mixture of coumarin 343 (**8**, 399 g, 1.40 mmol), compound **13** (460 mg, 2.10 mmol), and DIPEA (0.49 mL, 362 mg, 2.80 mmol) was dissolved in dry CH_2_Cl_2_ (15 mL). HATU (532 mg, 1.40 mmol) was added and the solution was stirred at rt for 17 h. The solvent was evaporated, the crude product was suspended in ethyl acetate and washed with 10% KHSO_4_ (3 × 40 mL), saturated NaHCO_3_ solution (3 × 40 mL) and brine (40 mL). The organic phase was dried over Na_2_SO_4_, filtrated, and evaporated in vacuo. The residue was purified by column chromatography using ethyl acetate as eluent to obtain compound **14** as an orange oil (600 mg, 1.23 mmol, 88%); ^1^H-NMR (500 MHz, DMSO-*d*_6_) δ 8.77 (t, *J* = 5.5 Hz, 1H, NH), 8.49 (s, 1H, 9-H), 7.22 (s, 1H, 8-H), 3.98 (s, 2H, OCH_2_CO), 3.61–3.54 (m, 4H), 3.53 (t, *J* = 5.6 Hz, 2H), 3.45 (q, *J* = 5.7 Hz, 2H, NHCH_2_CH_2_O, NHCH_2_CH_2_O, OCH_2_CH_2_O, OCH_2_CH_2_O), 3.34–3.29 (m, 4H, N(CH_2_CH_2_CH_2_)_2_), 2.73–2.68 (m, 4H, N(CH_2_CH_2_CH_2_)_2_), 1.89–1.85 (m, 4H, N(CH_2_CH_2_CH_2_)_2_), 1.40 (s, 9H, C(CH_3_)_3_); ^13^C-NMR (126 MHz, DMSO-d_6_) δ 169.4, 162.6, 162.0, 152.2, 148.1, 147.6, 127.2, 119.5, 108.0, 107.5, 104.7, 80.7, 70.0, 69.7, 69.2, 68.3, 49.7, 49.1, 38.9, 27.9, 26.9, 20.7, 19.7; LC-MS (ESI) (60% H_2_O to 100% MeOH in 10 min, then 100% MeOH over 10 min, DAD 200–500 nm), 95% purity, *m*/*z* = 487.2 ([M+H]^+^).

*Ebselen-coumarin Heterodimer* (**15**). Compound **14** (292 mg, 0.6 mmol) was dissolved in dry CH_2_Cl_2_(30 mL) and trifluoroacetic acid (6 mL) was added. The resulting reaction mixture was stirred at rt for 2 h. The solvent was then evaporated, and the residue was diluted with CH_2_Cl_2_(4 × 10 mL) and evaporated to remove the excess of trifluoroacetic acid. The crude product was dissolved in dry DMF (10 mL), and HATU (255 mg, 0.67 mmol) and DIPEA (0.70 mL, 517 mg, 4 mmol) were added. After stirring at room temperature for 30 min, compound **6** (334 mg, 0.80 mmol) was added and the reaction mixture was stirred at rt for 48 h. The solvent was then evaporated and the resulting residue was redissolved in DMF (3 mL). To this solution silica gel was added and the solvent was again evaporated. The crude product attached to silica gel was added to a column and purified using CH_2_Cl_2_/MeOH (9:1) as eluent. The product-containing fractions were combined and evaporated to dryness. MeOH (4 mL) was added, and the resulting precipitate was filtrated, washed with MeOH (2 × 5 mL) and washed with ethyl acetate (2 × 5 mL) to yield compound **15** as a yellow solid (50 mg, 12%); m.p. 120–123 °C; ^1^H-NMR (500 MHz, DMSO-*d*_6_) δ 8.81 (t, *J* = 5.5 Hz, 1H, CH_2_N*H*CO), 8.46 (s, 1H, coumarin 9-H), 8.19 (t, *J* = 6.2 Hz, 1H, CH_2_N*H*CO), 8.05 (d, *J* = 8.0 Hz, 1H, ebselen 4-H), 7.86 (dd, *J* = 7.8, 1.4 Hz, 1H, ebselen 7-H), 7.68–7.63 (m, 1H, ebselen Ar-H), 7.56–7.49 (m, 2H, phenylene 2-H, 6-H), 7.46 (t, *J* = 7.4 Hz, 1H, ebselen Ar-H), 7.33–7.27 (m, 2H, phenylene 3-H, 5-H), 7.17 (s, 1H, coumarin 8-H), 4.34 (d, *J* = 6.3 Hz, 2H, phenylene-C*H*_2_NHCO), 3.97 (s, 2H, OCH_2_CO), 3.66–3.59 (m, 4H), 3.55 (t, *J* = 5.5 Hz, 2H), 3.45 (q, *J* = 5.5 Hz, 2H, NHC*H*_2_CH_2_O, NHCH_2_C*H*_2_O, OC*H*_2_CH_2_O, OCH_2_C*H*_2_O), 3.30–3.24 (m, 4H, N(C*H*_2_CH_2_CH_2_)_2_), 2.67–2.63 (m, 4H, N(CH_2_CH_2_C*H*_2_)_2_), 1.84–1.80 (m, 4H, N(CH_2_C*H*_2_CH_2_)_2_); ^13^C-NMR (126 MHz, DMSO-*d*_6_) δ 169.4, 165.0, 162.6, 162.1 152.2, 148.1, 147.6, 138.9, 138.4, 137.2 132.3, 128.6, 128.0, 127.2, 126.3, 125.9, 124.5, 119.5, 107.9, 107.5, 104.7, 70.5, 70.2, 69.5, 69.2, 49.6, 49.1, 41.4, 38.9, 26.9, 20.6, 19.7, 19.7; LC-MS(ESI) (90% H_2_O to 100% MeOH in 10 min, then 100% MeOH over 10 min, DAD 200–500 nm), 89% purity, *m/z* = 715.2, 717.2 ([M+H]^+^); HRMS (ESI): *m*/*z* [M+H]^+^) calcd. for C_36_H_36_N_4_O_7_Se: 715.1830, 717.1822; found: 715.1750, 717.1750.

### 3.3. Modified Enzymatic Assays

*Cathepsin B.* Human isolated cathepsin B (Calbiochem, Darmstadt, Germany) was assayed spectrophotometrically (Cary 50 Bio, Varian, Agilent Technologies, Santa Clara, CA, USA) at 405 nm and at 37 °C. Assay buffer was 100 mM sodium phosphate buffer pH 6.0, 100 mM NaCl, 5 mM EDTA, 0.01% Brij 35. To 2 μL an enzyme stock solution of 1.81 mg/mL in 20 mM sodium acetate buffer pH 5.0 and 1 mM EDTA, a volume of 9.98 μL assay buffer containing 5 mM DTT was added. Then, 988.02 μL of assay buffer was added. This enzyme solution was incubated for 30 min at 37 °C. Stock solutions (10 mM) of ebselen and **15** were prepared in DMSO. A 100 mM stock solution of the chromogenic substrate Z-Arg-Arg-pNA was prepared with DMSO. The final concentration of DMSO was 5%, the final concentration of the substrate was 500 μM, and the final DTT concentration was 3.5 μM. Assays were performed with a final concentration of 253 ng/mL of cathepsin B. Into a cuvette containing 176 μL assay buffer, 7 μL DMSO, 1 μL of the substrate solution and 14 μL of the enzyme solution were added, thoroughly mixed, and incubated for 10 min at 37 °C. The reaction was initiated by adding 2 μL of DMSO or inhibitor solution and followed over 30 min.

*Cathepsin* L. Human isolated cathepsin L (Enzo Life Sciences, Lörrach, Germany) was assayed spectrophotometrically (Cary 50 Bio, Varian) at 405 nm and at 37 °C. Assay buffer was 100 mM sodium phosphate buffer pH 6.0, 100 mM NaCl, 5 mM EDTA, and 0.01% Brij 35. To 10 μL of an enzyme stock solution of 135 μg/mL in 20 mM malonate buffer pH 5.5, 400 mM NaCl, and 1 mM EDTA, a volume of 10 μL assay buffer containing 5 mM DTT was added. Then, 980 μL of assay buffer was added. This enzyme solution was incubated for 30 min at 37 °C. Stock solutions (10 mM) of ebselen and **15** were prepared in DMSO. A 10 mM stock solution of the chromogenic substrate Z-Phe-Arg-pNA was prepared with DMSO. The final concentration of DMSO was 5%, the final concentration of the substrate was 100 μM, and the final DTT concentration was 3.5 μM. Assays were performed with a final concentration of 94.5 ng/mL of cathepsin L. Into a cuvette containing 176 μL assay buffer, 6 μL DMSO, 2 μL of the substrate solution and 14 μL of the enzyme solution were added, thoroughly mixed, and incubated for 10 min at 37 °C. The reaction was initiated by adding 2 μL of DMSO or inhibitor solution and followed over 30 min.

## Figures and Tables

**Table 1:** Absorption and emission maxima of compounds **9** and **15**.

Compd.	Absorption			Emission		
	Buffer, pH 7.8	MeOH	CH_2_Cl_2_	Buffer, pH 7.8	MeOH	CH_2_Cl_2_
**9**	434 nm	436 nm	435 nm	494 nm	484 nm	470 nm
**15**	450 nm	436 nm	435 nm	494 nm	484 nm	470 nm

## Supplementary Material

^1^H-NMR and ^13^C-NMR spectra are available online at http://www.mdpi.com/1424-8247/9/3/43/s1.

## Abbreviations

The following abbreviations are used in this manuscript:

DIPEA	ethyldiisopropylamine
DTT	dithiothreitol
GPx	glutathione peroxidase
HAUT	1-[bis(dimethylamino)methylene]-1*H*-1,2,3-triazolo[4,5-*b*]pyridinium 3-oxid hexafluorophosphate
PMT	photomultiplier tube
TrxR	thioredoxin reductase
